# An insight into tissue culture-induced variation origin shared between anther culture-derived triticale regenerants

**DOI:** 10.1186/s12870-023-04679-w

**Published:** 2024-01-11

**Authors:** Renata Orłowska, Janusz Zimny, Jacek Zebrowski, Piotr Androsiuk, Piotr T. Bednarek

**Affiliations:** 1 Plant Breeding and Acclimatization Institute, National Research Institute, Radzików, Błonie, 05-870 Poland; 2 Institute of Biotechnology, College of Natural Science, University of Rzeszow, Al. Rejtana 16c, Rzeszow, 35-959 Poland; 3 Department of Plant Physiology, Genetics and Biotechnology, Faculty of Biology and Biotechnology, University of Warmia and Mazury in Olsztyn, Olsztyn, 10-719 Poland

**Keywords:** Anther culture, Triticale, Low methylated pectins, GSH, SAM, Copper ions, DNA methylation, DNA sequence, Green plant regeneration efficiency, Structural equation modeling

## Abstract

**Background:**

The development of the plant in vitro techniques has brought about the variation identified in regenerants known as somaclonal or tissue culture-induced variation (TCIV). S-adenosyl-L-methionine (SAM), glutathione (GSH), low methylated pectins (LMP), and Cu(II) ions may be implicated in green plant regeneration efficiency (GPRE) and TCIV, according to studies in barley (*Hordeum vulgare* L.) and partially in triticale (× *Triticosecale* spp. Wittmack ex A. Camus 1927). Using structural equation models (SEM), these metabolites have been connected to the metabolic pathways (Krebs and Yang cycles, glycolysis, transsulfuration), but not for triticale. Using metabolomic and (epi)genetic data, the study sought to develop a triticale regeneration efficiency statistical model. The culture’s induction medium was supplemented with various quantities of Cu(II) and Ag(I) ions for regeneration. The period of plant regeneration has also changed. The donor plant, anther-derived regenerants, and metAFLP were utilized to analyze TCIV concerning DNA in symmetric (CG, CHG) and asymmetric (CHH) sequence contexts. Attenuated Total Reflectance–Fourier Transfer Infrared (ATR-FTIR) spectroscopy was used to gather the metabolomic information on LMP, SAM, and GSH. To frame the data, a structural equation model was employed.

**Results:**

According to metAFLP analysis, the average sequence change in the CHH context was 8.65%, and 0.58% was *de novo* methylation. Absorbances of FTIR spectra in regions specific for LMP, SAM, and GSH were used as variables values introduced to the SEM model. The average number of green regenerants per 100 plated anthers was 2.55.

**Conclusions:**

The amounts of pectin demethylation, SAM, *de novo* methylation, and GSH are connected in the model to explain GPRE. By altering the concentration of Cu(II) ions in the medium, which influences the amount of pectin, triticale’s GPRE can be increased.

**Supplementary Information:**

The online version contains supplementary material available at 10.1186/s12870-023-04679-w.

## Background

With the development of in vitro tissue culture techniques, morphologically atypical regenerants representing somaclonal variation (or tissue culture-induced variation, TCIV) were noticed [1]. Some authors have questioned the phenomenon, claiming that the variation was caused by the so-called preexisting variation related to differences between individual cells of the donor plant [2]. It was simple to eliminate unusual regenerants that displayed unexpected morphological variation during culture, but somaclonal variation was contentious and has been the subject of ongoing debate [3–6]. DNA marker-based studies have shown that TCIV is linked to mutations [7], but some have indicated that the link is not clear [8] or was not found [9]. Some data suggested the significance of DNA methylation changes [10]. Furthermore, the relationship between components (sequence variation, DNA demethylation and *de novo* DNA methylation) of TCIV and green plant regeneration efficiency (GPRE) from anther cultures was proposed [11]. One of the biggest problems with TCIV and GPRE was that there needed to be more tightly defined biological materials to eliminate or lessen the effect of the preexisting variation [12]. Moreover, to reduce the amount of variation induced during doubled haploid donor plant regeneration it was suggested utilizing the generative progeny of double-haploid donor plants [13]. However, in some species, more than a single generative cycle following doubled haploid production may be required [10, 14]. It was also hard to find a DNA-based marker system that could quantify both changes in DNA methylation patterns and differences in sequences between regenerants and the donor plant simultaneously. The restriction was successfully overcome with the creation of the metAFLP methodology [15].

With the growing number of studies, it became apparent that the TCIV is a complex phenomenon involving DNA methylation within a variety of sequence contexts, including symmetric (CG, CHG) and asymmetric (CHH) sequences, and that the methylation of these sites differs in their epigenetic background [16–18]. It was shown that the DNA methylation pattern and the level of sequence variation induced *de novo* during tissue culture plant regeneration (androgenesis [13, 19] and embryogenesis [20, 21]) depend on species. It was also suggested that methylation may participate in GPRE [22]. Unfortunately, studies of TCIV failed to explain the nature of the phenomenon (for more information on this, see the reviews [23, 24]). Therefore, it was hypothesized that it might have a biochemical background. A series of studies conducted on barley and triticale demonstrated that S-adenosyl-L-methionine (SAM) [25], glutathione (GSH) [26], *b*-glucans [27]/pectins [28], and Cu(II) ions [22] are involved in GPRE. The metabolites, *via* structural equation models [28], were linked to the Krebs and Yang cycles and biochemical pathways, including glycolysis or transsulfuration. However, no generalized scheme linking the metabolome, TCIV and GPRE has been evaluated, including triticale.

We hypothesize that GPRE and TCIV have biochemical backgrounds linked to glycolysis, the Krebs and Yang cycles, and the transsulfuration pathway that influences GSH production and affects GPRE. Propper functioning of glycolysis during plant regeneration *via* anther culture may require copper ions as they act as cofactors of many enzymatic reactions. However, the relationships between metabolome, TCIV, and GPRE in triticale must be made apparent and should be investigated.

The study aimed to evaluate a statistical model of anther culture plant regeneration efficiency utilizing metabolomic data reflecting biochemical processes linked to DNA sequence and methylation changes induced *via in vitro* cultures of triticale. Such a model is required to progress in theoretical understanding and future practical knowledge utilization to increase GPRE in cereals.

## Results

A single double haploid plant’s generative progeny was chosen randomly, providing the explant tissue. Thirty-seven morphologically consistent regenerants that were identical to the donor plant were created by the eight experiments (A–H). The Cu(II), and Ag(I) ion concentrations in the induction medium (IM) and the length of the in vitro anther cultures differed between these eight investigations. There were anywhere between three and ten regenerants in each trial. Trial C had the lowest GPRE value, whereas trial H had the highest (Additional file 1; Tab S1). The fourteen-days-old leaf of a donor and leaves from the 37 regenerants were employed to extract the genomic DNA of sufficient quality and quantity for the metAFLP analysis. Quantitative metAFLP analysis of banding patterns shared by the donor plant and its regenerants revealed that the presence of sequence variation within the CHH asymmetric sequence context ranged from 8.48 to 8.91%, with the mean value equaling 8.65%. The CHH context’s *de novo* methylation ranged from 0.36 to 0.76% (mean value 0.58%) (Additional file 1; Tab S1).

The FTIR analysis delivered spectra previously assigned to metabolites (GSH: 2550 − 2540 cm^−1^ [26], SAM: 1630…1470 cm^−1^ [25]), representing biochemical pathways and cycles, and proved to be essential for partial structural equation models. The mean absorbance values of low methylated pectins (LMP), SAM, and GSH across all samples encompassing all trials were 0.5512, 3.9617, and 0.00515, respectively. All trials’ mean green plant regeneration efficiency was 2.55 and varied from 0.71 to 6.06. Minor standard errors, standard deviations, and variances were calculated for all variables across all samples (Additional file 1: Tab S1).

Based on 37 regenerants from eight experimental trials, the structural equation modeling analysis (Table 1) revealed a minor departure from the normal distribution, as shown by skewness and kurtosis values. All of the variables were quantitative and met the requirements of the Lindeberg-Lévy theorem [29], stating that if the formation of a variable of natural origin is influenced by a large number of primarily random factors, then the distribution of this variable is asymptotically convergent to the normal distribution. Thus, the variables’ asymptotic distribution is anticipated to approach their theoretical normal distribution.

**Table 1:** Descriptive statistics of the variables present in the postulated models

		Minimum	Maximum	Mean	Std. Deviation	Variance	Skewness	Kurtosis
Cu(II)		0.1	10.0	5.4270	3.5759	12.787	-0.102	-1.008
Ag(I)		0.0	60.0	22.4324	26.7089	713.363	0.704	-1.480
Time		35.0	49.0	43.7027	5.8112	33.770	-0.495	-1.371
CHH_DNMV		0.3600	0.7600	0.5838	0.1370	0.019	-0.194	-1.054
CHH_SV		8.4800	8.9100	8.6546	0.1146	0.013	-0.079	-0.754
990.950 cm^−1^ (LMP)		0.3583	0.9473	0.5512	0.1223	0.015	0.951	1.406
1630…1470 cm^−1^ (SAM)		2.6528	4.6522	3.9617	0.4635	0.215	-0.709	0.278
2550 − 2540 cm^−1^ (GSH)		0.0041	0.0060	0.0051	0.0004	0.000	-0.351	0.054
GPRE		0.7140	6.0610	2.5563	1.6589	2.752	0.932	-0.122

The 990.950 cm^−1^ reflects continuous FTIR spectrum starting from 990 and ending at 950 and encompasses values calculated every ten units, the range can be assigned to low methylated pectins (LMP); similarly the 2550 − 2540 cm^−1^ FTIR reflects data related only to the 10 units range and is assigned to glutathione (GSH), whereas the 1630…1470 cm^−1^ FTIR range encompasses combined spectra from the given range but the data is not continuous (some ranges were not included into combined variable) [28], the range is assigned to S-adenosyl-L-methionine (SAM)

Pearson’s linear correlation coefficients (Table 2) showed that Cu (II) was positively correlated with GPRE and FTIR GSH range, while a negative correlation was evaluated for CHH_SV. Ag(I) was negatively related to CHH_DNMV, whereas the time of anther culture was positively related to CHH_SV and negatively related to FTIR LMP range. CHH_DNMV was positively correlated with the FTIR SAM range. A positive correlation was also evaluated between FTIR SAM and FTIR GSH range. Furthermore, the FTIR GSH range was positively correlated with GPRE. The other correlations were insignificant.

**Table 2:** Pearson’s linear correlation coefficients for analyzed variables

	Cu(II)	Ag(I)	Time	CHH_DNMV	CHH_SV	FTIR spectra absorbance			GPRE
						990..950 cm^-1^ (LMP)	1630…1470 cm^-1^ (SAM)	2550-2540 cm^-1^ (GSH)	
Cu(II)	1								
Ag(I)	-0.181	1							
Time	-0.312	-0.203	1						
CHH_DNMV	0.320	-,426^**^	-0.028	1					
CHH_SV	-,386^*^	0.050	,607^**^	0.247	1				
990..950 cm^-1^ (LMP)	0.009	0.252	-,451^**^	0.039	-0.183	1			
1630...1470 cm^-1^ (SAM)	0.075	-0.020	-0.116	,389^*^	0.080	,388^*^	1		
2550-2540 cm^-1^ (GSH)	,385^*^	-0.229	0.081	0.171	-0.132	0.137	,650^**^	1	
GPRE	,807^**^	-0.201	-0.061	0.005	-0.297	-0.117	-0.052	,461^**^	1

*Correlation is significant at the 0.05 level (2-tailed)

**Correlation is significant at the 0.01 level (2-tailed)

**Fig. 1:**
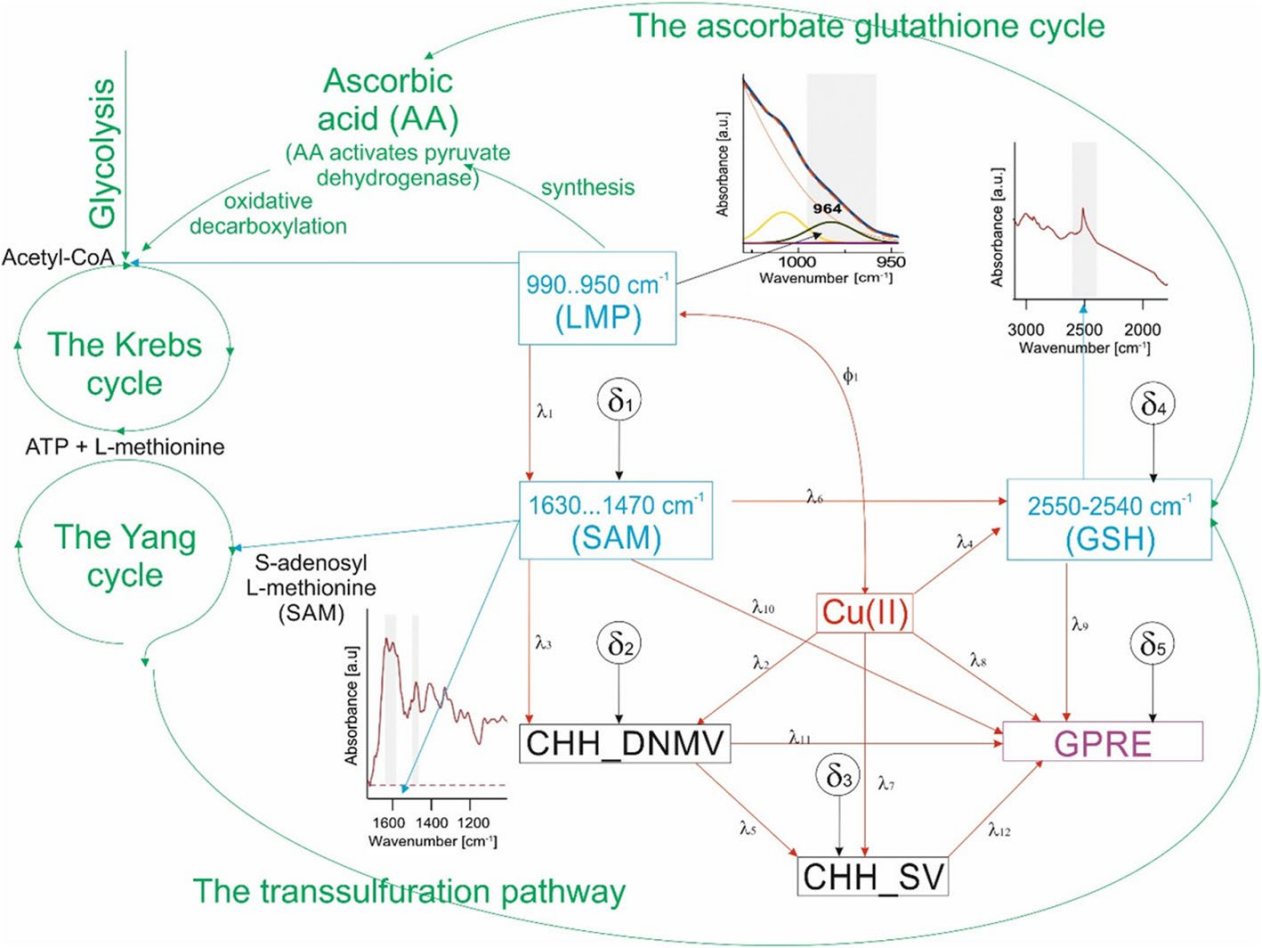
The schematic illustration of relationships between the biochemical pathways and cycles and the hypothesized SEM model, along with FTIR spectra bands specific for the metabolomic compounds included in the model The hypothesized SEM model with variables Cu(II), CHH_DNMV, CHH_SV, the FTIR absorbances at: 990.950 cm^−1^ assigned to low methylated pectins (LMP), 1630…1470 cm^−1^ assigned to S-adenosyl-L-methionine (SAM), and 2550 − 2540 cm^−1^ assigned to glutathione (GSH) as a function of GPRE (in purple). Red arrows indicate the SEM paths, whereas metabolites in the SEM are indicated by blue fonts and by blue arrows, indicating their position in biochemical paths and cycles and respective FTIR bands. The action of pectins *via* ascorbic acid (AA) on the tricarboxylic acid cycle (TCA) / Krebs cycle and the involvement of AA in the ascorbate-glutathione cycle, as well as other paths and cycles are given in green. Cu(II) (in red) denotes the concentration of copper ions (M) in the IM. GPRE stands for green plant regeneration efficiency (number of regenerants per 100 plated anthers). CHH_SV and CHH_DNMV are the metAFLP quantitative characteristics of sequence variation and *de novo* DNA methylation variation affecting CHH sequence contexts (in the dark), respectively *λ *_*1*_* -λ *_*12*_ path coefficients, *δ *_*1*_* -δ *_*5*_ residuals (experimental errors)

Data from previously published partial structural models of the relationships between CHH_SV, CHH_DNMV, Cu(II), Ag(I), time of anther cultures, *β*-glucans/pectins, GSH, and GPRE were employed (Ag^+^ [30], GSH [26], SAM [25], GSH and SAM [28]). Five endogenous variables (described by the model; CHH_DNMV, CHH_SV, FTIR absorbance in the region assigned to SAM, FTIR absorbance in the region assigned to GSH and GPRE) and two exogenous (not described within the model) (FTIR absorbance in the region assigned to LMP, Cu(II)) variables make up the proposed model. The relationships had a single covariance. All paths were non-recursive. There were five residuals in the model (Fig. 1).

Chi-square statistics analysis of the model fit revealed that the model was not significant (Table 3). A low *χ* ^*2*^ value indicates a well-fitting model when the sample size is big. However, the test may produce inaccurate results when the sample size is limited (as in the case of our data) [31]. In such a situation, it is advised to employ the chi-square test as the sole information criterion [32]. Thus, other models’ descriptive goodness-of-fit measures were applied. The model fits perfectly the experimental data, as evidenced by the *χ* ^*2*^/df being less than 3, the Root Mean Square Residuals (RMR) being under 0.05, and the Root Mean Square Error of Approximation (RMSEA) being below 0.05. The RMSEA was also within the 90% confidence interval. The Standardized RMR (SRMR) was < 0.08, indicating that the model was well-fitted. Except for the Adjusted Goodness-of-Fit Index (AGFI) and Relative Fit Index (RFI), which were slightly below the 0.9 cut-off values [33], nearly all goodness-of-fit measures appeared to match the proposed requirements (above 0.95 but not below 0.9). The fact that the Parsimonious Normed Fit Index (PNFI) and Parsimonious Comparative Fit Index (PCFI) were so modest suggests model complexity. The Expected Cross-Validation Index (ECVI) is within the 90% confidence interval, indicating that the data should be repeatable using the same sample size. Last but not least, Hoelter’s 0.05 index was 67, indicating that at least 67 samples needed to be utilized in the SEM design in order to create a model with a *p* = 0.05 significance (Table 3).

**Table 3:** Summary of the analyzed structural equation model

Indices	Statistics
*Chi-squared (χ)*^2^	*8.4558*
*p*	*0.3903*
*χ *^2^*/df*	*1.057*
RMR / Root mean square residuals	0.0264
GFI / Goodness-of-fit index	0.944
AGFI / Adjusted goodness-of-fit index	0.8039
PGFI / Parsimony GFI	0.2697
NFI / Normed fit index	0.9319
RFI / Relative fit index	0.8213
IFI / Incremental fit index	0.9961
TLI / Tucker-Lewis Index	0.9884
CFI / Comparative fit index	0.9956
PNFI / Parsimonious normed fit index	0.381
PCFI / Parsimonious comparative fit index	0.355
RMSEA / Root mean square error of approximation	0.0379
RMSEA-LO90 / Lower boundary of a 90% confidence interval of the REMSEA	0
RMSEA-HI90 / Higher boundary of a 90% confidence interval of the REMSEA	0.2021
PCLOSE / P-value of the null hypothesis	0.4581
SRMR / Standardized RMR	0.0719
ECVI / Expected Cross-Validation Index	1.346
ECVI-L090 / Lower boundary of a 90% confidence interval of the ECVI	1.33
ECVI-HI90 / Higher boundary of a 90% confidence interval of the REMSEA	1.66
Hoelter’s Critical N (0.05)	67

All the paths’ (*β*) coefficients (except CHH_SV→GPRE) of the hypothesized model were significant (Table 4). The effect of Cu(II) on GPRE was the highest and most positive, followed by the FTIR SAM range on the FTIR GSH range as indicated by standardized estimates. The effect of Cu(II) on CHH_SV was the next one but negative. The effects between CHH_DNMV and CHH_SV, FTIR GSH range and GPRE, FTIR LMP range and FTIR SAM range, and Cu (II) and FTIR GSH range were also strong and positive. The effects of the FTIR SAM range and CHH_DNMV on GPRE were moderately strong and negative. The only covariate relationship between Cu(II) and FTIR LMP range was insignificant.

**Table 4:** Path coefficients, variances and covariances for the analyzed model

Parameters	Effects			Estimates (b)	SE	Test statistics	Standardized estimate (*β*)
*Path coefficients*							
*λ*_*1*_	990..950 cm^-1^ (LMP)	**⟶**	1630…1470 cm^-1^ (SAM)	1.4717	0.582	2.5287^*^	0.3884
*λ*_*2*_	Cu(II)	** ⟶**	CHH_DNMV	0.0112	0.0056	2.0019^*^	0.2947
*λ*_*3*_	1630…1470 cm^-1^ (SAM)	** ⟶**	CHH_DNMV	0.1084	0.0432	2.5118^*^	0.3697
*λ*_*4*_	Cu(II)	** ⟶**	2550-2540 cm^-1^ (GSH)	0	0	2.9726^**^	0.3437
*λ*_*5*_	CHH_DNMV	** ⟶**	CHH_SV	0.3457	0.123	2.8115^**^	0.4082
*λ*_*6*_	1630…1470 cm^-1^ (SAM)	** ⟶**	2550-2540 cm^-1^ (GSH)	0.0006	0.0001	5.4822^***^	0.6339
*λ*_*7*_	Cu(II)	** ⟶**	CHH_SV	-0.0166	0.0047	-3.5252^***^	-0.5158
*λ*_*8*_	Cu(II)	** ⟶**	GPRE	0.3795	0.0471	8.0586^***^	0.8252
*λ*_*9*_	2550-2540 cm^-1^ (GSH)	** ⟶**	GPRE	1535.381	438.3667	3.5025^***^	0.3908
*λ*_*10*_	1630…1470 cm^-1^ SAM)	** ⟶**	GPRE	-1.0138	0.4032	-2.5143^*^	-0.2858
*λ*_*11*_	CHH_DNMV	** ⟶**	GPRE	-3.0603	1.1604	-2.6373^**^	-0.2530
*λ*_*12*_	CHH_SV	** ⟶**	GPRE	2.2539	1.2656	1.7809	0.1578
*Covariances*							
	Cu(II)	**⟵⟶ **	990..950 cm^-1^ (LMP)	0.0038	0.0709	0.0536	
*Variances*							
*δ*_*1*_				0.1775	0.0418	4.2426^***^	
*δ*_*2*_				0.0139	0.0033	4.2426^***^	
*δ*_*3*_				0	0	4.2426^***^	
*δ*_*4*_				0.0089	0.0021	4.2426^***^	
*δ*_*5*_				0.5115	0.1206	4.2426^***^	
Cu(II)				12.4414	2.9325	4.2426^***^	
990..950 cm^-1^ (LMP)				0.0146	0.0034	4.2426^***^	

^*^ — significant at *p* ≤ 0.05; ^**^ — significant at *p* ≤ 0.01; ^***^ — significant at *p* ≤ 0.001

The hypothesized model includes direct, indirect, and total effects (Table 5). Direct effects indicate the direct impact of variables on each other. Indirect effects, however, indicate the mediation effect of variables mediating the described relationship and may also indicate relationships of variables for which no direct effects were observed. The GPRE variable showed the most significant dependence on Cu (II) (total *β* = 0.8226; including the direct effect as *β* = 0.8252 and negative and small indirect effect). The FTIR GSH range was the second and the most influential variable with a positive direct effect on GPRE (*β* = 0.3908). The CHH_DNMV, and FTIR SAM range affected GPRE preferentially *via* negative direct effects; however, indirect effects were positive. Finally, the FTIR LMP range affected GPRE *via* small, negative and indirect effects. FTIR GSH range was affected mainly by the FTIR SAM range with a positive direct effect (*β* = 0.6339). It was also directly and positively controlled by Cu(II) and indirectly by LMP (*β* = 0.3437 and *β* = 0.2462, respectively). CHH_SV was negatively dependent on Cu (II) (primarily *via* direct (*β* = -0.5158) effect with total equal *β* = -0.3955) but positively dependent on CHH_DNMV *(via* direct and total effect (*β* = -0.4082). CHH_DNMV depended on the FTIR SAM range *via* direct effect (*β* = 0.3697) followed by Cu(II) (*β* = 0.2947) and by FTIR LMP range *via* positive indirect effect (*β* = 0.1436). Lastly, the FTIR SAM range was directly affected by the level of pectin demethylation (*β* = 0.3884). The effect was positive.

**Table 5:** Direct, indirect and total effectsss for the analyzed model

Effect			Estimates (*b*)			Standardized Estimates (*β*)		
			Direct Effect	Indirect Effect	Total Effect	Direct Effect	Indirect Effect	Total Effect
GPRE								
990..950 cm^-1^ (LMP)	→	GPRE	-	-0.5628	-0.5628	-	-0.0419	-0.0419
Cu(II)	→	GPRE	0.3795	-0.0012	0.3782	0.8252	-0.0026	0.8226
1630…1470 cm^-1^ (SAM)	→	GPRE	-1.0138	0.6314	-0.3824	-0.2858	0.178	-0.1078
CHH_DNMV	→	GPRE	-3.0603	0.7791	-2.2811	-0.2530	0.0644	-0.1886
CHH_SV	→	GPRE	2.253sss9	-	2.2539	0.1578	-	0.1578
2550-2540 cm^-1^ (GSH)	→	GPRE	1535.381	-	1535.381	0.3908	-	0.3908
2550-2540 cm^-1^ (GSH)								
990..950 cm^-1^ (LMP)	→	2550.2540 cm^-1^ (GSH)	-	0.0008	0.0008	-	0.2462	0.2462
Cu(II)	→	2550.2540 cm^-1^ (GSH)	-	-	-	0.3437	-	0.3437
1630…1470 cm^-1^ (SAM)	→	2550.2540 cm^-1^ (GSH)	0.0006	-	0.0006	0.6339	-	0.6339
CHH_SV								
990..950 cm^-1^ (LMP)	→	CHH_SV	-	0.0552	0.0552	-	0.0586	0.0586
Cu(II)	→	CHH_SV	-0.0166	0.0039	-0.0127	-0.5158	0.1203	-0.3955
1630…1470 cm^-1^ (SAM)	→	CHH_SV	-	0.0375	0.0375	-	0.1509	0.1509
CHH_DNMV	→	CHH_SV	0.3457	-	0.3457	0.4082	-	0.4082
CHH_DNMV								
990..950 cm^-1^ (LMP)	→	CHH_DNMV	-	0.1546	0.1546	-	0.1436	0.1436
Cu(II)	→	CHH_DNMV	0.0112	-	0.0112	0.2947	-	0.2947
1630…1470 cm^-1^ (SAM)	→	CHH_DNMV	0.1084	-	0.1084	0.3697	-	0.3697
1630…1470 cm^-1^ (SAM)								
990.950 cm^-1^ (LMP)	→	1630…1470 cm^-1^	1.4717	-	1.4717	0.3884	-	0.3884

The Ag(I) failed to show significant effects when GSH was assumed (the characteristic was not included in the model), despite being significant in our previous model describing similar relationships but without GSH [30]. We also failed to include the Time of another culture in the model.

## Discussion

A complex statistical method known as “structural equation modeling” was used to test the working hypotheses that in vitro tissue culture conditions affect anther tissue culture plant regeneration and biochemical pathways and cycles and that the biochemical background regulates GPRE, which is under Cu(II) ions’ control acting as a cofactor in enzymatic reactions. In biology, the reflection of theoretical assumptions is often known, and the effects of specific variables in a model are usually big. Because of this, the sample size is less critical than in psychology, where only minor effects are usually assumed. Such a situation was present in the current study, where the model was constructed based on 37 samples and included many variables. Different aspects of the final model were tested to overcome obvious statistical limitations.

We recently used the SEM method to build models that explain TCIV or GPRE. It was shown that sequence variation is linked to DNA methylation status change [22] and that S-adenosyl-L-methionine, a compound made from ATP and L-methionine, is involved in the process. ATP synthesis requires copper ions in the Krebs cycle’s electron transfer chain complex IV, which links the TCA cycle with the Yang cycle. SAM is a crucial molecule the cell uses for methylating other cellular compounds [34]. So, the model showed that SAM is involved in TCIV. Furthermore, it shows that changes in the CHH sequence context of DNA methylation play a role in sequence variation and GPRE. Evidence demonstrates that GSH may be critical in green plant regeneration efficiency [26]. Its importance was demonstrated experimentally in the rye (*Secale cereale* L.) and triticale [35, 36]. Using SEM, we discovered close relationships between GSH and GPRE, allowing us to include metabolite variables in the SEM model, demonstrating that the Yang cycle disturbances influence GSH synthesis *via* the transsulfuration pathway [34, 37].

Furthermore, we have also suggested that the Krebs cycle may also be affected by in vitro tissue culture manipulation [28]. It was demonstrated that glycolysis could use *β*-glucans as a carbon source to pump the barley’s TCA cycle [38]. A comparable study in triticale yielded inconclusive results [28]. Nevertheless, the study suggested that pectin demethylation level could indirectly affect TCA *via* acetyl-CoA. Pectins partly affect ascorbic acid (AA) synthesis, and AA turns on pyruvate dehydrogenase, which is part of the pyruvate dehydrogenase complex. The latter is responsible for pyruvate’s oxidative decarboxylation, leading to acetyl-CoA used by the Krebs cycle. Following the reasoning, we have tested the relationships between the FTIR signal from low methylated pectins, the TCA, and the Yang cycles. Thus, distinct but closely linked biochemical background aspects of the TCIV and GPRE work in concert [28].

The study’s main finding was the importance of Cu(II) in affecting GPRE and sequence variation. Cu(II) can cause DNA modifications via oxidizing methylated cytosines, resulting in point mutations [39]. Cu(II) may also work with Zn/Cu-dependent dismutase to remove excess ROS [40] that can cause DNA mutations. Since several SEMs showed links between different parts of TCIV and GPRE, we found combining all models into a single scheme justified, considering that tissue culture experiments only looked at a small number of regenerants.

The fact that CHH_DNMV adversely influences GPRE may indicate that gene expression regulation affects the phenomenon and that CHH_DNMV may even block GPRE. The concept is consistent with earlier research showing that sequence variation impacting the CHH context may have had little to no effect on GPRE. Contrarily, the impact of the CHH on GPRE comes from the interaction of methylation cytosines with Cu (II). Pectin’s demethylation level indirectly affects SAM modulating DNA *de novo* methylation, which is consistent with the model’s biochemical background. Pectins are also linked to GSH *via* the ascorbate-glutathione cycle. However, we did not test whether or how much ascorbic acid (AA) synthesis is affected by the in vitro culture conditions. Still, considering the role of GSH and the Krebs cycle involved in the GPRE and TCIV, AA is an integral part of the phenomenon that links oxidative processes. The model demonstrates that SV is only one of several minor issues affecting GPRE. However, DNA methylation changes should still be considered because they may control how much a gene is expressed. However, the fact that changes in DNA methylation have a negligible effect on GPRE may mean that problems with biochemical cycles and pathways are more important. The provided model has practical ramifications in addition to its scientific significance. It shows that adjusting the concentration of copper ions in induction media (IM) can modify cell activity and increase GPRE.

Our previous studies have demonstrated that SAM and GSH impact GPRE. SAM achieves this by methylating the asymmetric CHH sequence, which is related to *de novo* methylation [25]. At the same time, GSH does so through an epigenetic process that is not fully understood [26]. *β*-glucans may be the primary source of glucose during tissue culture carbon shortages [27] and may, therefore, supply energy to glycolysis, thereby affecting the Krebs cycle. Similarly, pectins are involved in the production of ascorbic acid and may indirectly affect the Krebs cycle via acetyl-CoA. Moreover, pectins are the most likely metabolites involved in the interactions between SAM, GSH, Cu(II), and GPRE [28]. Lastly, we cannot ignore the positive impact of copper ions, which help elevate GPRE levels [28]. The current model includes LMP, and its significance is based on the significance of the former SEMs. Unfortunately, with a limited sample size (limited number of regenerants), we could not build such an advanced model without demonstrating that all preceding SEMs were significant. Still, our results show that the model explaining GPRE is not complete. We hypothesize that some unrecognized or unconsidered enzymatic processes or epigenetic regulation of gene expression that result in GPRE possibly involving Cu(II) as a cofactor should be taken into account. The vast and robust impact of GSH on GPRE and the effect of SAM on GSH lend some support to this idea.

## Conclusions

The structural equation model shown is based on biochemical cycles and pathways involving the metabolites involved in TCIV, GPRE, and epigenetic mechanisms. The model explains GPRE by connecting LMP, SAM, *de novo* methylation and GSH. The model, which is still being worked on, suggests possible directions for more research. First, it is crucial to investigate the function of antioxidants such as ascorbic acid. Second, it’s critical to analyze whether gene expression varies due to varying culture conditions and assess the potential functions of the genes. The SEM approach is also helpful because it gives a framework and a reason to change other cultural conditions to improve GPRE and control TCIV. Our findings also clarify GSH’s importance. A fascinating question is how LMP and SAM are involved in TCIV and GPRE. It is possible to raise the GPRE in triticale by adjusting Cu(II) ion concentrations in the IM and manipulating pectins. Additionally, it is essential to look into how different metal ions behave in the IM and how to optimize their concentration.

## Methods

The current study was based on former experiments involving specially designed biological materials [13], the metAFLP approach [19], FTIR data concerning *β*-glucans/pectins [27, 28], SAM [25], and GSH [26]. A structural equation model [30] was used to frame the data.

A donor plant is a generative offspring of a double haploid in the biological system. In order to regenerate new plants under various Cu(II), Ag(I) ion concentrations in the induction medium (IM) and time of anther culture (covers period starting from the plating of anthers on the IM to the collection of calli, embryo-like structures, or somatic embryos, and their transfer on regeneration media), the donor plant functioned as a source of explant tissues (anthers) (Additional file 1: Tab S1). Eight different A-H trial conditions were prepared to test the effect of Cu(II), Ag(I) ions, and time of anther cultures on the tissue culture-induced variation and the number of green regenerants obtained. Green plant regeneration efficiency was calculated for each trial based on the estimated number of green regenerants per 100 plated anthers. The donor plant and its regenerants were assessed for the following physical features (plant growth, leaf shape, color and width, tillering mode, and spikes number) [13].

The unique properties of the isoschizomers *Acc65*I and *Kpn*I, which have varying sensitivity to the site’s DNA methylation pattern and its surroundings, are the foundation of the metAFLP approach. Using two metAFLP platforms (*Acc65*I/*Mse*I and *Kpn*I/*Mse*I) for the amplification of DNAs from a donor plant and its regenerants, the method enables quantification of sequence and DNA methylation events that could be standardized, resulting in respective variations.

Samples for the mid-infrared spectroscopy were lyophilized and homogenized into powder (ball mill, MM 400, Retsch, Haan, Germany). Nicolet iN10 MX infrared imaging microscope (Thermo Fisher Scientific, Waltham, Massachusetts, USA), equipped with a deuterated triglycine sulphate (DTGS) detector and a KBr beam splitter, was used for the measurements. Using the one-bounce diamond crystal and the ATR accessory, 64 spectra per sample were obtained in the Attenuated Total Reflectance (ATR) mode with a 4 cm^−1^ resolution in the wavenumber range between 600 and 4000 cm^−1^ (Smart Orbit, Thermo Scientific, Madison, WI, USA). Before each measurement, the diamond crystal’s surface was cleaned with water or propanol to get rid of any leftovers from previous samples. OMNIC software was used to record, average, and baseline-correct the spectra (v. 9.0, Thermo Fischer Scientific Inc.). The ChemoSpec [41] package of the R programming language [42] used to accomplish the normalization to the unit area inside the 1800 − 900 cm^−1^ wavenumber region, statistics (mean, SD), and printing the spectra.

In order to build a theoretical illustration of green plant regeneration efficiency using AMOS [43] under SPSS v. 28 [44] software, metAFLP data evaluated based on the donor plant and its regenerants as well as the absorbance of the FTIR spectra bands assigned to LMP, SAM, and GSH were incorporated in a structural equation model.

### Supplementary Information

**Additional file 1: Table S1.** The arrangement of *in vitro* tissue culture conditions. Description of data: Concentrations of Cu (II) and Ag (I) ions in the induction medium (IM), time of anther cultures (days) and *de novo* methylation of the CHH sequence contexts (CHH_DNMV), sequence variation within the CHH context (CHH_SV), low methylated pectins (LMP), S-adenosyl-L-methionine (SAM), glutathione (GSH), and green plant regeneration efficiency (GPRE) for all trials (A-H). *De novo* methylation of CHH sequence contexts (CHH_DNMV), sequence variation within CHH contexts (CHH_SV), pectin, S-adenosyl-L-methionine (SAM), glutathione (GSH) and green plant regeneration efficiency (GPRE) for all samples (A-H), depending on the arrangement of *in vitro* tissue culture conditions, such as Cu (II) and Ag (I) ion concentrations in induction medium (IM), time of anther culture (days). The 990..950 cm^-1^ reflects continuous FTIR spectrum starting from 990 and ending at 950 and encompasses values calculated every ten units; similarly the 2550-2540 cm^-1^ FTIR reflects data related only to the 10 units range, whereas the 1630…1470 cm^-1^ FTIR range encompasses combined spectra from the given range but the data is not continuous (some ranges were not included into combined variable)

## Data Availability

The datasets used and/or analysed during the current study are available from the corresponding author on reasonable request.

## References

[1] Eeckhaut T, Van Houtven W, Bruznican S, Leus L, Van Huylenbroeck J (2020). Somaclonal variation in Chrysanthemum × morifolium protoplast regenerants. Front Plant Sci.

[2] Evans D, Sharp A, Medina-Filho WR (1984). Somaclonal and gametoclonal variation. Am J Bot.

[3] Ferreira MS, Rocha AJ, Nascimento FS, Oliveira WDS, Soares JMS, Rebouças TA, Morais Lino LS, Haddad F, Ferreira CF, Santos-Serejo JA (2023). The role of somaclonal variation in plant genetic improvement: a systematic review. Agronomy.

[4] Ghosh A, Igamberdiev AU, Debnath SC (2021). Tissue culture-induced DNA methylation in crop plants: a review. Mol Biol Rep.

[5] Krishna H, Alizadeh M, Singh D, Singh U, Chauhan N, Eftekhari M, Sadh RK (2016). Somaclonal variations and their applications in horticultural crops improvement. 3 Biotech.

[6] Neelakandan AK, Wang K (2012). Recent progress in the understanding of tissue culture-induced genome level changes in plants and potential applications. Plant Cell Rep.

[7] Hou B-H, Tsai Y-H, Chiang M-H, Tsao S-M, Huang S-H, Chao C-P, Chen H-M (2022). Cultivar-specific markers, mutations, and chimerisim of Cavendish banana somaclonal variants resistant to Fusarium oxysporum f. sp. cubense tropical race 4. BMC Genomics.

[8] Metakovsky EV, Novoselskaya AY, Sozinov AA (1987). Problems of interpreting results obtained in studies of somaclonal variation in gliadin proteins in wheat. Theor Appl Genet.

[9] Tikendra L, Potshangbam AM, Dey A, Devi TR, Sahoo MR, Nongdam P (2021). RAPD, ISSR, and SCoT markers based genetic stability assessment of micropropagated Dendrobium fimbriatum Lindl. Var. oculatum hk. f.- an important endangered orchid. Physiol Mol Biol Plants.

[10] Machczyńska J, Orłowska R, Mańkowski DR, Zimny J, Bednarek PT (2014). DNA methylation changes in triticale due to in vitro culture plant regeneration and consecutive reproduction. Planr Cell Tissue Organ Cult.

[11] Orłowska R (2022). Triticale doubled haploid plant regeneration factors linked by structural equation modeling. J Appl Genet.

[12] Matsuda S, Sato M, Ohno S, Yang S-J, Doi M, Hosokawa M (2014). Cutting leaves and plant growth regulator application enhance somaclonal variation induced by transposition of *VGs1* of *Saintpaulia*. J Japanese Soc Hortic Sci.

[13] Pachota KA, Orłowska R, Bednarek PT (2022). Medium composition affects the tissue culture-induced variation in triticale regenerants. Planr Cell Tissue Organ Cult.

[14] Orłowska R, Machczyńska J, Oleszczuk S, Zimny J, Bednarek PT (2016). DNA methylation changes and TE activity induced in tissue cultures of barley (*Hordeum vulgare* L). J Biol Res (Thessaloniki Greece).

[15] Bednarek PT, Orłowska R, Koebner RM, Zimny J (2007). Quantification of the tissue-culture induced variation in barley (*Hordeum vulgare* L). BMC Plant Biol.

[16] Fang J, Jiang J, Leichter SM, Liu J, Biswal M, Khudaverdyan N, Zhong X, Song J (2022). Mechanistic basis for maintenance of CHG DNA methylation in plants. Nat Commun.

[17] Gallego-Bartolomé J (2020). DNA methylation in plants: mechanisms and tools for targeted manipulation. New Phytol.

[18] Muyle AM, Seymour DK, Lv Y, Huettel B, Gaut BS (2022). Gene body methylation in plants: mechanisms, functions, and important implications for understanding evolutionary processes. Genome Biol Evol.

[19] Orłowska R, Bednarek PT (2020). Precise evaluation of tissue culture-induced variation during optimisation of in vitro regeneration regime in barley. Plant Mol Biol.

[20] Orłowska R, Zimny J, Bednarek PT (2021). Copper ions induce DNA sequence variation in zygotic embryo culture-derived barley regenerants. Front Plant Sci.

[21] Pachota KA, Orłowska R (2022). Effect of copper and silver ions on sequence and DNA methylation changes in triticale regenerants gained via somatic embryogenesis. J Appl Genet.

[22] Orłowska R, Pachota KA, Androsiuk P, Bednarek PT (2022). Triticale green plant regeneration is due to DNA methylation and sequence changes affecting distinct sequence contexts in the presence of copper ions in induction medium. Cells.

[23] Bednarek PT, Orłowska R (2020). Plant tissue culture environment as a switch-key of (epi)genetic changes. PCTOC.

[24] Bednarek PT, Pachota KA, Dynkowska WM, Machczynska J, Orłowska R (2021). Understanding in vitro tissue culture-induced variation phenomenon in microspore system. Int J Mol Sci.

[25] Orłowska R, Zebrowski J, Zimny J, Androsiuk P, Bednarek PT (2022). S-Adenosyl-L-methionine and Cu(II) impact green plant regeneration efficiency. Cells.

[26] Bednarek PT, Orłowska R, Mańkowski DR, Zimny J, Kowalczyk K, Nowak M, Zebrowski J (2022). Glutathione and copper ions as critical factors of green plant regeneration efficiency of triticale in vitro anther culture. Front Plant Sci.

[27] Bednarek PT, Zebrowski J, Orłowska R (2020). Exploring the biochemical origin of DNA sequence variation in barley plants regenerated via in vitro anther culture. Int J Mol Sci.

[28] Orłowska R, Zebrowski J, Dynkowska WM, Androsiuk P, Bednarek PT (2023). Metabolomic changes as key factors of green plant regeneration efficiency of triticale in vitro anther culture. Cells.

[29] Taboga M (2012). Lectures on probability theory and mathematical statistics.

[30] Bednarek PT, Orłowska R, Mańkowski DR, Oleszczuk S, Zebrowski J (2021). Structural equation modeling (SEM) analysis of sequence variation and green plant regeneration via anther culture in barley. Cells.

[31] Kenny DA, McCoach DB (2003). Effect of the number of variables on measures of fit in structural equation modeling. Struct Equation Modeling: Multidisciplinary J.

[32] MacCallum RC, Browne MW, Sugawara HM (1996). Power analysis and determination of sample size for covariance structure modeling. Psychol Methods.

[33] Mulaik SA, James LR, Van Alstine J, Bennett N, Lind S, Stilwell CD (1989). Evaluation of goodness-of-fit indices for structural equation models. Psychol Bull.

[34] Watanabe M, Chiba Y, Hirai MY (2021). Metabolism and regulatory functions of O-Acetylserine, S-Adenosylmethionine, homocysteine, and serine in plant development and environmental responses. Front Plant Sci.

[35] Żur I, Dubas E, Krzewska M, Zieliński K, Fodor J, Janowiak F (2019). Glutathione provides antioxidative defence and promotes microspore-derived embryo development in isolated microspore cultures of triticale (× Triticosecale Wittm). Plant Cell Rep.

[36] Zieliński K, Krzewska M, Żur I, Juzoń K, Kopeć P, Nowicka A, Moravčiková J, Skrzypek E, Dubas E (2020). The effect of glutathione and mannitol on androgenesis in anther and isolated microspore cultures of rye (*Secale cereale* L). Planr Cell Tissue Organ Cult.

[37] Mäkinen K, De S (2019). The significance of methionine cycle enzymes in plant virus Infections. Curr Opin Plant Biol.

[38] Roulin S, Buchala AJ, Fincher GB (2002). Induction of (1→3,1→4)-β-D-glucan hydrolases in leaves of dark-incubated barley seedlings. Planta.

[39] Atha DH, Wang H, Petersen EJ, Cleveland D, Holbrook RD, Jaruga P, Dizdaroglu M, Xing B, Nelson BC (2012). Copper oxide nanoparticle mediated DNA damage in terrestrial plant models. Environ Sci Technol.

[40] Berwal MK, Ram C. Superoxide Dismutase: A Stable Biochemical Marker for Abiotic Stress Tolerance in Higher Plants. In: *Abiotic and Biotic Stress in Plants.* 2018. p. 541–628. Oliveira ABd (Series Editor).

[41] Hanson BA. ChemoSpec: Exploratory Chemometrics for Spectroscopy. R package version 4.4.97. DePauw University: Greencastle; 2017. Available online: https://CRAN.R-project.org/package=ChemoSpec. Accessed 12 Dec 2017.

[42] R Core Team: R. A Language and Environment for Statistical Computing; R Foundation for Statistical Computing: Vienna, Austria. Available online: http://www.R-project.org . Accessed 31 Mar 2021. 2021.

[43] Arbuckle JL (2014). Amos. Version 27.0 edition. pp. computer program.

[44] IBM SPSS Statistics for Windows. Vers. 28.0.0. Amronk, NY: IBMCorp; 2021.

