# Supplementary material for: An insight into tissue culture-induced variation origin shared between anther culture-derived triticale regenerants
Source: BMC Plant Biol. 2024 Jan 11;24:43. doi: 10.1186/s12870-023-04679-w (PMC10782687; doi:10.1186/s12870-023-04679-w)
Supplement: Supplementary file 1 — Additional file 1: Table S1. The arrangement of in vitro tissue culture conditions. Description of data: Concentrations of Cu (II) and Ag (I) ions in the induction medium (IM), time of anther cultures (days) and de novo methylation of the CHH sequence contexts (CHH_DNMV), sequence variation within the CHH context (CHH_SV), low methylated pectins (LMP), S-adenosyl-L-methionine (SAM), glutathione (GSH), and green plant regeneration efficiency (GPRE) for all trials (A-H). De novo methylation of CHH sequence contexts (CHH_DNMV), sequence variation within CHH contexts (CHH_SV), pectin, S-adenosyl-L-methionine (SAM), glutathione (GSH) and green plant regeneration efficiency (GPRE) for all samples (A-H), depending on the arrangement of in vitro tissue culture conditions, such as Cu (II) and Ag (I) ion concentrations in induction medium (IM), time of anther culture (days). The 990..950 cm-1 reflects continuous FTIR spectrum starting from 990 and ending at 950 and encompasses values calculated every ten units; similarly the 2550-2540 cm-1 FTIR reflects data related only to the 10 units range, whereas the 1630…1470 cm-1 FTIR range encompasses combined spectra from the given range but the data is not continuous (some ranges were not included into combined variable) [file 12870_2023_4679_MOESM1_ESM.docx]

File name: Additional file 1

File format: DOC (Microsoft Word)

Title of data: Table S1 The arrangement of *in vitro* tissue culture conditions

Description of data: Concentrations of Cu (II) and Ag (I) ions in the induction medium (IM), time of anther cultures (days) and *de novo* methylation of the CHH sequence contexts (CHH_DNMV), sequence variation within the CHH context (CHH_SV), low methylated pectins (LMP), S-adenosyl-L-methionine (SAM), glutathione (GSH), and green plant regeneration efficiency (GPRE) for all trials (A-H). *De novo* methylation of CHH sequence contexts (CHH_DNMV), sequence variation within CHH contexts (CHH_SV), pectin, S-adenosyl-L-methionine (SAM), glutathione (GSH) and green plant regeneration efficiency (GPRE) for all samples (A-H), depending on the arrangement of *in vitro* tissue culture conditions, such as Cu (II) and Ag (I) ion concentrations in induction medium (IM), time of anther culture (days). The 990..950 cm^-1^ reflects continuous FTIR spectrum starting from 990 and ending at 950 and encompasses values calculated every ten units; similarly the 2550-2540 cm^-1^ FTIR reflects data related only to the 10 units range, whereas the 1630…1470 cm^-1^ FTIR range encompasses combined spectra from the given range but the data is not continuous (some ranges were not included into combined variable) .

| Trial | *In vitro* anther culture conditions | | | metAFLP quantitative characteristics (%) | | FTIR spectra absorbance | | | GPRE |
| --- | --- | --- | --- | --- | --- | --- | --- | --- | --- |
|  | Cu (II) (µM) | Ag (I) (µM) | Time (days) | CHH_SV | CHH_DNMV | 990..950 cm^-1^  (LMP) | 1630…1470 cm^-1^ (SAM) | 2550-2540 cm^-1^ (GSH) |  |
| A | 0.1 | 10 | 42 | 8.66 | 0.37 | 0.4244967 | 3.409978 | 0.004591 | 0.87 |
| A | 0.1 | 10 | 42 | 8.66 | 0.37 | 0.4601159 | 3.685812 | 0.00494 | 0.87 |
| A | 0.1 | 10 | 42 | 8.52 | 0.36 | 0.4290325 | 4.252169 | 0.005324 | 0.87 |
| B | 0.1 | 60 | 49 | 8.64 | 0.37 | 0.5362991 | 3.241198 | 0.004879 | 1.52 |
| B | 0.1 | 60 | 49 | 8.64 | 0.37 | 0.7048459 | 3.992293 | 0.00512 | 1.52 |
| B | 0.1 | 60 | 49 | 8.79 | 0.37 | 0.6337196 | 3.960656 | 0.005144 | 1.52 |
| B | 0.1 | 60 | 49 | 8.79 | 0.37 | 0.5405354 | 3.265786 | 0.004353 | 1.52 |
| B | 0.1 | 60 | 49 | 8.79 | 0.56 | 0.5241464 | 3.840676 | 0.004985 | 1.52 |
| C | 5 | 60 | 42 | 8.76 | 0.75 | 0.4898344 | 4.324086 | 0.004768 | 0.71 |
| C | 5 | 60 | 42 | 8.79 | 0.56 | 0.5163602 | 4.262209 | 0.005038 | 0.71 |
| C | 5 | 60 | 42 | 8.64 | 0.55 | 0.3952185 | 3.350444 | 0.00412 | 0.71 |
| D | 5 | 0 | 49 | 8.64 | 0.55 | 0.5949863 | 4.341778 | 0.005176 | 2.38 |
| D | 5 | 0 | 49 | 8.64 | 0.55 | 0.5419134 | 4.221504 | 0.005264 | 2.38 |
| D | 5 | 0 | 49 | 8.76 | 0.75 | 0.3582991 | 3.972691 | 0.005502 | 2.38 |
| D | 5 | 0 | 49 | 8.76 | 0.75 | 0.6118391 | 4.603247 | 0.00604 | 2.38 |
| D | 5 | 0 | 49 | 8.76 | 0.75 | 0.4799925 | 4.017514 | 0.005451 | 2.38 |
| D | 5 | 0 | 49 | 8.76 | 0.75 | 0.4125308 | 3.988978 | 0.005293 | 2.38 |
| D | 5 | 0 | 49 | 8.76 | 0.75 | 0.4807893 | 4.260563 | 0.005133 | 2.38 |
| D | 5 | 0 | 49 | 8.76 | 0.75 | 0.5455717 | 4.614823 | 0.00552 | 2.38 |
| D | 5 | 0 | 49 | 8.76 | 0.75 | 0.559616 | 4.183686 | 0.004793 | 2.38 |
| D | 5 | 0 | 49 | 8.91 | 0.76 | 0.7138336 | 4.546406 | 0.005358 | 2.38 |
| E | 5 | 10 | 35 | 8.63 | 0.73 | 0.6305158 | 4.241465 | 0.004881 | 1.17 |
| E | 5 | 10 | 35 | 8.63 | 0.73 | 0.5993996 | 3.521011 | 0.004362 | 1.17 |
| E | 5 | 10 | 35 | 8.48 | 0.72 | 0.700787 | 3.754181 | 0.004652 | 1.17 |
| E | 5 | 10 | 35 | 8.48 | 0.72 | 0.6240595 | 4.082022 | 0.005249 | 1.17 |
| E | 5 | 10 | 35 | 8.5 | 0.54 | 0.9472981 | 4.180354 | 0.005302 | 1.17 |
| F | 10 | 10 | 49 | 8.48 | 0.54 | 0.4278094 | 3.311297 | 0.005046 | 3.79 |
| F | 10 | 10 | 49 | 8.65 | 0.55 | 0.4314824 | 3.334939 | 0.005121 | 3.79 |
| F | 10 | 10 | 49 | 8.65 | 0.55 | 0.4707907 | 3.645356 | 0.005566 | 3.79 |
| G | 10 | 60 | 35 | 8.62 | 0.56 | 0.6980939 | 4.526688 | 0.005394 | 4.24 |
| G | 10 | 60 | 35 | 8.49 | 0.55 | 0.7079717 | 4.414281 | 0.005685 | 4.24 |
| G | 10 | 60 | 35 | 8.49 | 0.55 | 0.6860557 | 4.652246 | 0.005823 | 4.24 |
| G | 10 | 60 | 35 | 8.65 | 0.55 | 0.6611504 | 3.924192 | 0.005085 | 4.24 |
| H | 10 | 0 | 42 | 8.49 | 0.55 | 0.4560213 | 4.267821 | 0.005692 | 6.06 |
| H | 10 | 0 | 42 | 8.49 | 0.55 | 0.4444432 | 3.86516 | 0.005502 | 6.06 |
| H | 10 | 0 | 42 | 8.65 | 0.55 | 0.5139003 | 2.6528 | 0.004594 | 6.06 |
| H | 10 | 0 | 42 | 8.65 | 0.55 | 0.4403233 | 3.87212 | 0.005502 | 6.06 |
| Mean |  |  |  | 8.65 | 0.58 | 0.5511913 | 3.96169 | 0.005142 | 2.56 |
| SE |  |  |  | 0.02 | 0.02 | 0.02011 | 0.07620 | 0.00007 | 0.27 |
| SD |  |  |  | 0.11 | 0.14 | 0.12231 | 0.46350 | 0.00042 | 1.66 |
| Variance |  |  |  | 0.01 | 0.02 | 0.01496 | 0.21483 | 0.00000 | 2.75 |
